# Quantifying Carbon Losses Associated with Photorespiration and Drought Stress in Two Dominant Mediterranean Pine Species

**DOI:** 10.3390/plants15162527

**Published:** 2026-08-20

**Authors:** Emre Yazar, Bülent Akgün, Emre Babur

**Affiliations:** 1 Department of Bioengineering and Sciences, Graduate School of Natural and Applied Sciences, Kahramanmaraş Sütçü İmam University, 46050 Kahramanmaraş, Türkiye; emre.yazar6@gmail.com; 2 Department of Forest Engineering, Faculty of Forestry, Kahramanmaraş Sütçü İmam University, 46050 Kahramanmaraş, Türkiye

**Keywords:** *Pinus brutia*, *Pinus nigra*, photorespiration, drought stress, net primary productivity, carbon sequestration, Mediterranean forests, adaptive forest management, carbon-market additionality

## Abstract

Photorespiration and drought-induced stomatal closure are two important physiological constraints that reduce carbon assimilation and productivity in C_3_ forest trees under Mediterranean climatic conditions. Türkiye’s two dominant commercial pine species, *Pinus brutia* Ten. (Calabrian pine) and *Pinus nigra* J.F. Arnold subsp. *pallasiana* (Anatolian black pine), together cover approximately 8.15 million hectares. This study integrated published gas-exchange measurements, radiation-use efficiency estimates from MODIS, official forest inventory data, and dendrochronological growth records into a counterfactual accounting framework and propagated parameter uncertainty by Monte Carlo simulation (N = 40,000 draws). The two constraints jointly reduced weighted-mean net primary productivity (NPP) from a radiation-limited potential of 5.61 to an actual 3.46 Mg C ha^−1^ yr^−1^, a reduction of 37.9% (95% CI 32.2–43.4%). Decomposition shows that 47.7% of this loss is the obligate metabolic cost of C_3_ carboxylation, which no silvicultural intervention can address, while 52.3%—20.1 of the 37.9 percentage points—is drought-attributable. Nationally, the deficit corresponds to 64.3 Mt CO_2_ yr^−1^ of forgone sequestration (47.8–81.2) and 34.4 Mm^3^ yr^−1^ of forgone stemwood-volume equivalent (24.9–44.5), of which approximately 20.6 Mm^3^ would be merchantable, giving an annual economic deficit of USD 3.37 billion (2.40–6.48). Filtering the drought-attributable component for eligible area, recovery efficiency, additionality, leakage, and permanence yields approximately 1.0 Mt CO_2_ yr^−1^ of potentially issuable credits, fewer than two per cent of the headline figure. Eco-physiological suppression of this magnitude is currently invisible in national forest carbon accounting, and its recognition bears directly on dynamic baseline design and on the credibility of offsets generated from Mediterranean conifer forests.

## 1. Introduction

Roughly 30% of Türkiye’s land area is wooded, and the country’s 23.4 million hectares of forests support a significant rural economy and a large-scale national carbon pool [1,2,3]. Within this area, two pine species stand out in terms of their commercial and ecological importance: the first is *Pinus brutia* Ten. and the second is *Pinus nigra* J.F. Arnold subsp. *pallasiana* (Lamb.) Holmboe. Together, these two species occupy just over four out of every ten forested hectares in the country and supply the majority of the industrial roundwood that reaches domestic mills [4]. The growing-stock series compiled in the FAO Global Forest Resources Assessment 2015 ranks *P. nigra* first and *P. brutia* third by national volume, with 315 and 262 million m^3^, respectively, in 2010, placing both species above every other conifer recorded for the country [4].

The economic gravity of these two pines reflects more than simple area dominance. *P. brutia*, often described as the keystone conifer of the northeastern Mediterranean, blankets roughly 30,000 km^2^ of natural stands, largely along the seaward flank of the Taurus Mountains [5,6,7]. *P. nigra* subsp. *pallasiana*, with about 4.2 Mha of stands [8], climbs higher up the elevation gradient and pushes deeper into the continental interior than *P. brutia* ever does, marking the upper montane belt of the western Taurus and the steppe-forest transition of inner Anatolia.

There is, however, an awkward physiological truth behind the timber figures: the realized productivity of both species sits well below what their site potential would allow. Two processes are jointly responsible. The first is photorespiration, the unavoidable side-reaction in which RuBisCO fixes O_2_ rather than CO_2_, releasing previously assimilated carbon and consuming additional ATP and NADPH in the recycling pathway [9,10,11]. The second is the closure of stomata under water deficit; protective in hydraulic terms, this response also curtails CO_2_ supply to the mesophyll and forces a steep decline in net assimilation [12,13,14]. The two mechanisms are not independent: a stomatal-driven fall in intercellular CO_2_ concentration (C_i_) simultaneously favors the oxygenize reaction of RuBisCO, so the carbon-loss penalty from stomatal limitation includes an additional photorespiratory tax that operates in the same physiological window [10,11].

In Türkiye’s Mediterranean and sub-Mediterranean climates, these two processes act in concert for several weeks every summer. Mid-day canopy temperatures of 35–40 °C, vapor-pressure deficits often above 4 kPa, and soil-moisture minima in July-August collectively yield a paradoxical phenology in which the months of highest solar radiation are also the months of lowest carbon uptake [13,15,16]. The physiological literature has documented this pattern in detail at the leaf and stand scale [14,15,17], and dendrochronological work has confirmed its imprint on radial growth across both species [18,19]. What has not yet been attempted is an explicit, economically calibrated quantification of the carbon—and therefore the timber and the sequestration—that these two processes represent as a latent loss to the national carbon and timber accounts.

The novelty of this study lies in the coupling rather than in any single component. Leaf-level photorespiration and stomatal limitation are well characterized; ecoregion-scale productivity is mapped; national forest area and round wood statistics are published; and carbon and timber prices are observable. What has not been conducted is to carry a physiological constraint through all four of these scales in one internally consistent accounting framework and to express the result in the units in which forest and climate policy is actually written. Doing so converts a leaf-level biochemical property into a national inventory quantity, and it is that conversion, rather than any new measurement, that this paper contributes.

This study therefore pursues four objectives. First, to describe the spatial production system of *P. brutia* and *P. nigra* at the ecological-region scale (Section 2). Second, to synthesize the published physiological evidence into zone-specific fractional carbon losses attributable to photorespiration and to drought-induced stomatal limitation, with an auditable derivation and explicit uncertainty (Section 3 and Section 4.3). Thirdly, we will construct a counterfactual accounting framework that partitions the resulting deficit into obligate metabolic and drought-attributable components. We will also propagate parameter uncertainty through this framework (see Section 4 and Section 5). Fourth, to establish which portion of the deficit is addressable by silviculture and which portion, if any, could satisfy the eligibility requirements of a carbon market (Section 6). The significance of the exercise is that the two constraints are not marginal: they account for more than a third of the potential productivity of 8.15 Mha of commercially dominant forest, in a country building a domestic carbon-pricing framework against a net-zero commitment for 2053.

## 2. Production Systems of *Pinus brutia* and *Pinus nigra* in Türkiye

### 2.1. Ecological-Region Framework

**Figure 1:**
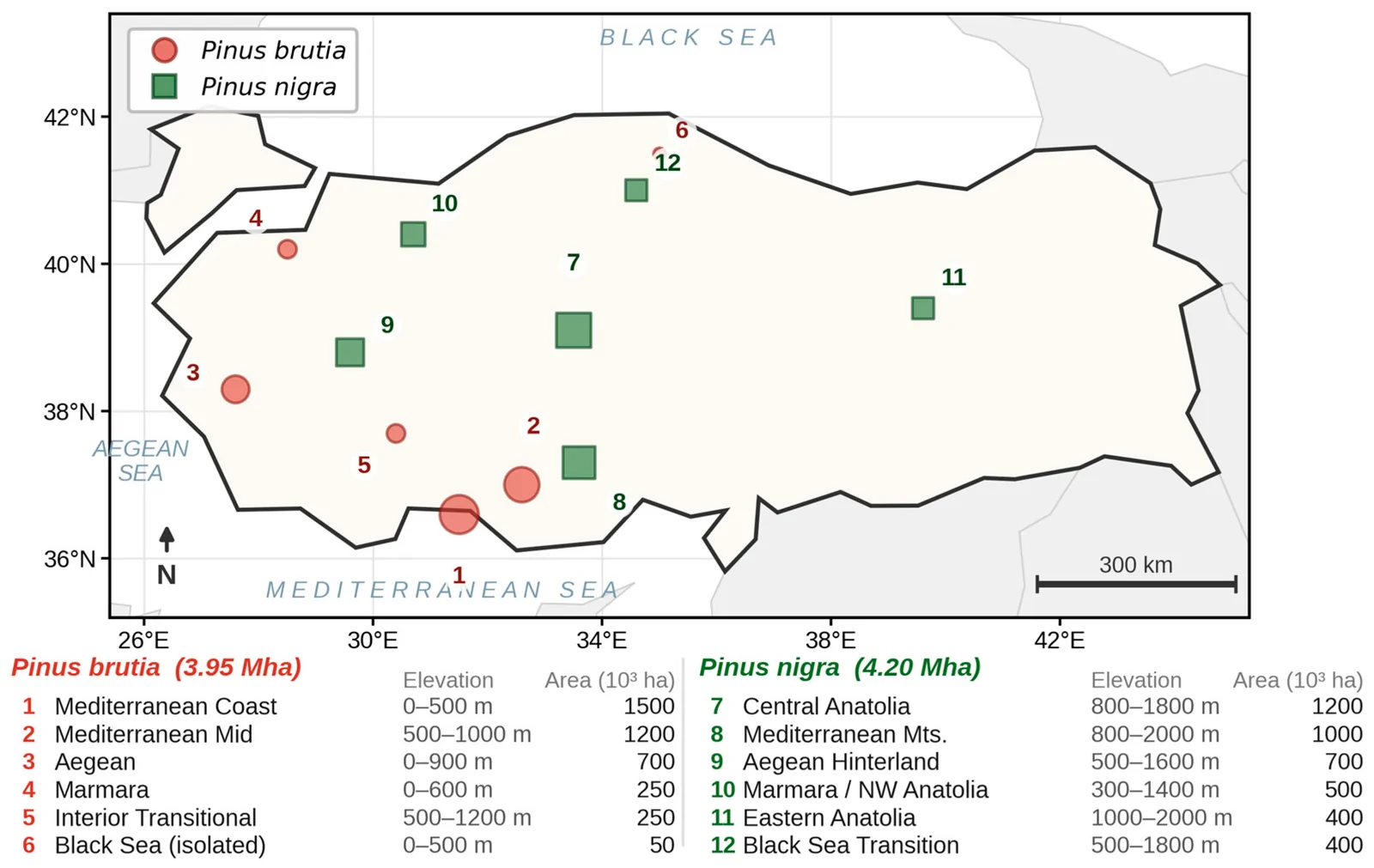
Distribution of the *P. brutia* and *P. nigra* production zones of Table 1 and Table 2 over the national outline of Türkiye. Circles denote *P. brutia* and squares *P. nigra*; symbol area is proportional to the standing area of each zone and symbols are plotted at indicative zone centroids. Numbers key to the panel below, which gives the elevation band and standing area of each zone. Base outline: Natural Earth 1:110 m. Zone areas and elevation bands after [1,2,5,8].

The Türkiye General Directorate of Forestry partitions the national forest estate across seven ecological regions: Mediterranean, Aegean, Marmara, Black Sea, Central Anatolia, Eastern Anatolia, and Southeastern Anatolia [2,8]. The regions differ enough in temperature seasonality, precipitation totals, and soil parent material that within-species productivity varies more across regions than between management classes within a single region [8,19]. Figure 1 places the most important production zones for each species on the administrative outline of Türkiye. The zone-level area, elevation, precipitation, and mean annual increment parameters that underlie this distribution are reported in Table 1 and Table 2 for *P. brutia* and *P. nigra*, respectively.

### 2.2. Pinus brutia—The Mediterranean Coastal Pine

*P. brutia* is overwhelmingly a species of the Mediterranean, Aegean, and (more marginally) Marmara regions. Survey work in the late 1980s mapped roughly 30,000 km^2^ of natural stands across the country, of which about 87% sit close to the Mediterranean coast, 10% occupy interior transitional sites, and a residual 3% cling to small areas near the Black Sea margin [5,6]. Vertically, the species spans sea level to roughly 1300 m, with its best stands clustered between about 500 and 800 m on north-facing slopes, where afternoon temperature and vapor-pressure deficit are partially buffered by aspect and proximity to the sea [3,5,7,17,19]. Mean annual increment at productive Mediterranean sites is typically reported at 3.5–6.2 m^3^ ha^−1^ yr^−1^ [19].

### 2.3. Pinus nigra—The Interior Montane Specialist

*P. nigra* subsp. *pallasiana* occupies a different envelope altogether. With approximately 4.2 Mha of stands [3,8,20], it is the most widespread native conifer in the country and the leading commercial species by national growing stock since 1990 [4]. Its core range covers the Central Anatolian highlands, the upper Taurus and Beydağları, the Aegean hinterland, and parts of Eastern Anatolia, with mixed stands at the lower margin commonly involving *P. brutia* and at the upper margin *Pinus sylvestris* L. and *Abies* spp. The species is markedly sensitive to spring-to-early-summer drought, especially in the Lakes District and along the steppe transition. Tree-ring chronologies from across western Anatolia consistently identify May-June precipitation as the principal limiting variable for radial growth, and the magnitude of that drought sensitivity is greater at the southern, lower-elevation margins of the range [18,19].

### 2.4. National Roundwood Production and the Contribution of the Two Pines

Türkiye’s harvested roundwood has expanded sharply since 2000. The national 2022 cut totaled approximately 25 million m^3^, composed of 9 Mm^3^ sawlogs, 9 Mm^3^ fibre and chip wood, 5 Mm^3^ paper wood, and 1 Mm^3^ industrial wood, while standing-timber sales reached 29 Mm^3^ in 2023 [1,2]. Pine species dominate the industrial roundwood stream. At the OGM pine-sawlog stumpage range of approximately USD 60–90 m^−3^ observed in the 2020–2023 auctions [2], the combined annual stumpage value attributable to *P. brutia* and *P. nigra* exceeds USD 500 million; including downstream conversion and processing multipliers, the sector-level contribution comfortably exceeds USD 1 billion per year [1].

## 3. Eco-Physiological Carbon-Loss Mechanisms

### 3.1. Photorespiration: An Unavoidable Warm-Season Tax

Every conifer in Türkiye uses C_3_ photosynthesis, and every C_3_ leaf pays a photorespiratory price. The enzyme RuBisCO is dual-substrate by design: it catalyses both the carboxylation of ribulose-1,5-bisphosphate (RuBP), the productive step, and its oxygenation, which initiates the glycolate cycle and releases previously fixed CO_2_ along with substantial ATP and NADPH consumption [9,10]. Two parameters control the carboxylation-to-oxygenation ratio: the CO_2_/O_2_ specificity factor of the enzyme (S*c/o*), which falls with temperature, and the intercellular CO_2_ concentration *C_i_*, which falls when stomata close [9,21,22]. The combined effect is non-linear: at 25 °C the photorespiratory CO_2_ release in a well-watered C_3_ leaf already amounts to roughly a quarter of the rate of net carboxylation [9,11]; at 30 °C in air with present-day CO_2_ the theoretical efficiency loss approaches 48% [11], and under combined heat and drought the published range for forest stands sits at 25–40% of GPP [10,11].

For *P. brutia* this physiology has a specific signature. Recent multi-site gas-exchange work across eastern-Mediterranean populations shows that atmospheric drought, quantified through VPD, exerts stronger control on maximum photosynthetic capacity than the land-surface moisture index, and that *P. brutia* at the highest VPD sites adopts a clearly conservative seasonal trajectory: photosynthetic activity is concentrated almost entirely in spring, with the summer period treated as a write-off [15]. The strategy is isohydric and prioritizes xylem safety [15,23], but it sacrifices most of the season’s incident radiation.

### 3.2. Stomatal Limitation Under Water Deficit

Stomatal closure is the second mechanism by which carbon assimilation falls during the Türkiye summer. As soil water declines and atmospheric VPD rises, guard cells lose turgor in an ABA-mediated response, lowering stomatal conductance (*g_s_*) and curtailing both transpiration and CO_2_ diffusion into the mesophyll [12,13,14]. In *Pinus sylvestris*, used here as a closely studied physiological analogue, net photosynthesis halts or turns negative once needle water potential falls below about −1.16 MPa [13]. For *P. nigra*, the consequences of severe summer drought are direct and well documented: declining trees exhibit lower non-structural carbohydrate stores, reduced xylem-conduit safety margins, and isotopic signatures (δ^13^C) indicative of suppressed stomatal conductance and reduced assimilation throughout the growing season [14,18,24]. Sap-flow studies in *P. brutia* confirm the same general pattern, with marked summer reduction in transpiration even in stands that are otherwise well established at the site [23,25].

**Figure 2:**
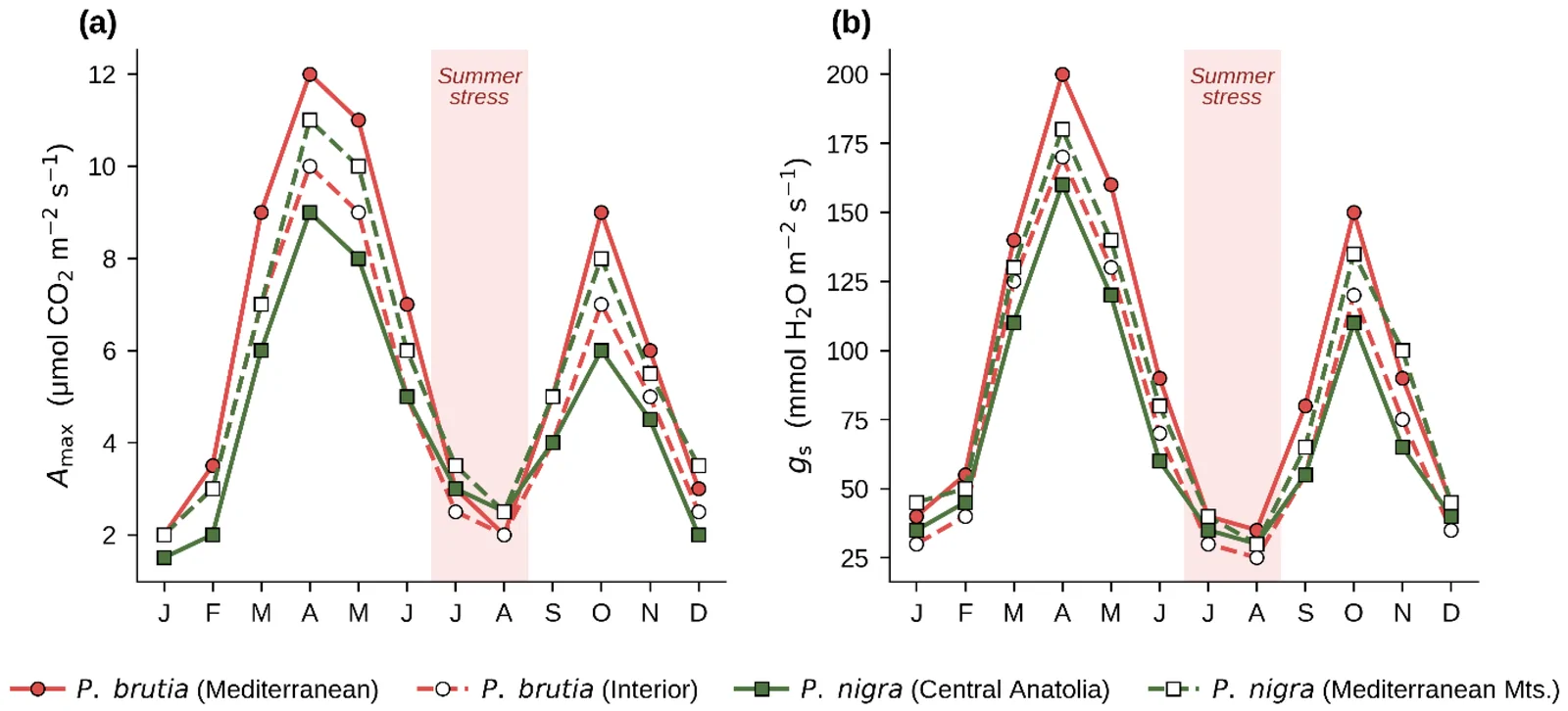
Seasonal phenology of (**a**) maximum photosynthetic capacity (A_max_) and (**b**) stomatal conductance (g_s_) for *P. brutia* and *P. nigra* in representative production zones. Curves are constructed from published gas-exchange ranges in Mediterranean and Anatolian field studies [14,15,17,26]. The shaded band marks the July-August summer-stress window, during which photorespiratory and stomatal effects act jointly.

A 2023 dendrometer study at a mixed Burdur *P. brutia*-*P. nigra* stand quantified the magnitude of summer-season carbon limitation directly through tree-water-deficit indicators: under matched VPD and soil-water-potential conditions, both species exhibited measurable stem-water deficits, with *P. nigra* showing the higher mean daily increment in the well-watered season and *P. brutia* maintaining the longer growth period overall [26]. Figure 2 summarizes the resulting bimodal phenology of *A*_max_ and *g_s_* for both species in two contrasting zones.

### 3.3. Process-Based Modelling and Integrated Stress Fractions

A spatially explicit, process-based forest-carbon model for Türkiye *P. brutia* stands, parameterized against long-term flux and tree-ring data spanning 1980–2020, reproduces the observed sensitivity of GPP, NPP, ecosystem respiration, and NEP to temperature and water along elevational gradients [16]. The same model shows that the realized photosynthetic optimum under Mediterranean summer water stress lies at a temperature lower than the theoretical biochemical optimum, because stomatal closure pulls the effective *T*_opt_ downward to prevent runaway water loss [16]. The implication is that the carbon loss from drought is not merely a *C_i_*-driven RuBisCO effect; it is also a mesophyll-diffusion and *T*_opt_-shift effect, and the three combine non-additively.

Converging gas-exchange, modelling, and dendroclimatic evidence allows zone-specific fractions of GPP lost to each mechanism to be written down (Table 3). Because stomatal closure simultaneously inflates photorespiration, the two losses are treated multiplicatively with a coupling coefficient λ, derived from first principles in Section 4.4. The derivation protocol for the fractions themselves is set out step by step in Section 4.3, and a per-zone audit trail is provided as Supplementary Table S1. The fractions are a structured elicitation bounded by published process constraints rather than a statistical estimation from primary data, and each therefore carries an explicit ±25% plausibility interval that is propagated through the uncertainty analysis of Section 5.5.

## 4. Counterfactual Modelling: A World Without Photorespiratory and Drought-Induced Carbon Constraints

### 4.1. Conceptual Framework and the Status of the Counterfactual

The counterfactual scenario asks the following: if photorespiration and drought-induced stomatal limitation were eliminated, holding all other biophysical constraints constant, what would be the realized NPP, timber volume, and economic output of Türkiye’s *P. brutia* and *P. nigra* forests over 2000–2023? The scenario is biologically unrealizable; it serves as an analytical instrument to isolate the economic cost of the two physiological constraints.

Two readings of the counterfactual must be kept apart. As an accounting quantity it measures the full physiological discount that separates realized productivity from radiation-limited potential, and it is in that sense that it bears on national carbon inventories and on the interpretation of forest sink estimates. As a management quantity it is not a target at all: the obligate photorespiratory component is a property of Rubisco and is unavailable to silviculture at any intensity of intervention. We therefore report the counterfactual gap in decomposed form throughout, and confine all management inference to the drought-attributable component identified in Section 5.1. The counterfactual is a benchmark against which the physiological discount can be measured, not a ceiling on what management could recover.

### 4.2. Model Structure and Governing Equations

The model operates in three linked modules. (i) A biophysical baseline computes NPP for each zone × species combination from radiation-use efficiency (RUE), absorbed PAR (APAR), photorespiratory and stomatal fractional losses, and a carbon-use efficiency (CUE) parameter. (ii) A counterfactual NPP is computed by relaxing both fractional losses. (iii) An economic translation converts the NPP difference into forgone stemwood volume and forgone CO_2_ sequestration, valued at contemporary stumpage and carbon prices.

For each zone × species unit *z*, potential gross primary productivity is the product of absorbed radiation and its potential conversion efficiency:

GPP_pot,z_ = APAR_z_ × RUE_pot,z_ (1)

The effective photorespiratory fraction combines the well-watered fraction and its drought amplification (Section 4.4):

*f*_PR,eff,z_ = *f*_PR,z_ (1 + λ *f*_ST,z_) (2)

The two losses act sequentially on the assimilation stream, so the combined fractional reduction is multiplicative rather than additive:

*f*_comb,z_ = 1 − (1 − *f*_PR,eff,z_)(1 − *f*_ST,z_) (3)

Actual and counterfactual net primary productivity follow directly, the counterfactual relaxing both fractions to zero:

NPP_act,z_ = GPP_pot,z_ (1 − *f*_comb,z_) × CUE  NPP_cf,z_ = GPP_pot,z_ × CUE (4)

The forgone assimilation is ΔNPP_z_ = NPP_cf,z_ − NPP_act,z_. National aggregates are obtained by area weighting over the twelve units, Σ_z_ *A*_z_ ΔNPP_z_, and all weighted means reported in Section 5 are area-weighted means of the zonal values rather than functions of the weighted-mean inputs. The distinction is not cosmetic: applying a weighted-mean loss fraction to a weighted-mean potential gives 3.48 Mg C ha^−1^ yr^−1^ for actual NPP, whereas the correct zonal aggregation gives 3.46, because loss fractions and potential productivity covary across a heterogeneous estate.

### 4.3. Derivation of the Zone-Specific Stress Fractions

The fractions in Table 3 are derived by a four-step protocol applied identically to all twelve zone × species combinations.

Step 1—Process bounds. The admissible range for the photorespiratory fraction is taken from the C_3_ process literature: photorespiratory CO_2_ release amounts to roughly one quarter of net carboxylation in a well-watered leaf at 25 °C [9,11]; the theoretical efficiency loss approaches 48% at 30 °C at present-day CO_2_ [11]; and the published range for forest canopies under combined heat and drought is 25–40% of GPP [10,11]. The admissible range for the stomatal fraction is bounded below by zones in which summer water deficit is not growth-limiting and bounded above by the assimilation suppression reported for Mediterranean pines during the July–August window [13,14,15].

Step 2—Zone thermal and evaporative scalar. Each zone is assigned a summer-stress index with three terms. The first is the elevation band, which sets the lapse-rate component of canopy temperature. The second is a maritime-buffering term: in zones adjacent to the Mediterranean, Aegean, Marmara, and Black Sea coasts, higher summer cloud cover and lower vapor-pressure deficit suppress the midday temperature maximum that drives the photorespiratory penalty, so elevation alone overstates the thermal load. The third is the depth of the July–August soil-moisture minimum rather than the mean annual total, because a zone can carry high annual precipitation and still experience a severe summer deficit; the Mediterranean Coast is the clearest such case. The index is normalized across the twelve zones and mapped linearly onto the Step-1 bounds. Supplementary Table S2 reports a diagnostic in which the second and third terms are omitted and shows that a mapping from elevation and mean annual precipitation alone recovers the stomatal fractions well but not the photorespiratory fractions; that result is the quantitative basis for including them.

Step 3—Species trajectory adjustment. The two species partition the stress differently and the mapping is adjusted accordingly. *P. brutia* at high-VPD sites follows an isohydric, spring-loaded trajectory in which assimilation is concentrated before the summer window and xylem safety is prioritized over carbon gain [15,23]; this raises the photorespiratory fraction relative to the stomatal fraction at low elevation, because the canopy remains illuminated and warm while assimilation is curtailed. *P. nigra* in the continental interior is limited principally by May–June precipitation [18] and, under severe drought, exhibits the hydraulic-failure and carbon-starvation signature described by Savi et al. [14]; this raises the stomatal fraction relative to the photorespiratory fraction in Central and Eastern Anatolia.

Step 4—Dendroclimatic consistency check. The resulting combined reduction for each zone is checked against the sign and rank order of the reported growth–climate sensitivities for the two species [18,19] and against the elevational gradient in simulated net ecosystem productivity for *P. brutia* [16]. Where the rank order conflicted, the Step-2 index was revised; no zone required adjustment beyond the Step-1 bounds.

We emphasize the epistemic status of these values. They are a structured expert elicitation constrained by published process bounds, not a statistical estimation from primary observations, because no study reports either fraction directly at the ecoregion scale for these species. That absence is itself part of what this synthesis is designed to expose. Table 3 therefore reports each fraction with a ±25% plausibility interval, these intervals are propagated through the Monte Carlo analysis of Section 5.5, and Section 6.4 identifies the paired gas-exchange and flux campaign that would replace elicitation with measurement.

### 4.4. Coupling of Photorespiratory and Stomatal Limitation

Photorespiratory CO_2_ release per gross carboxylation is set by the ratio of the CO_2_ compensation point in the absence of dark respiration, Γ*, to the chloroplastic CO_2_ concentration, *C*_c_ [9,27]. Stomatal closure amplifies this ratio by two multiplicative routes, and the coefficient λ of Equation (2) is the product of the two.

The diffusive route follows from the fact that assimilation is diffusion-limited under closure, so *C*_c_ must fall; the demand function *A*(*C*_c_) = (*C*_c_ − Γ*)/(*C*_c_ + *K*_m_) fixes how far it falls for a given reduction in assimilation, independently of whether the additional resistance is stomatal or mesophyll. The thermal route follows from the loss of latent cooling: needle temperature rises, and Γ* rises with it under the Bernacchi et al. temperature functions [9]. Evaluated at 28 °C with a 20% stomatal loss, the diffusive route requires *C*_c_ to fall from 193 to 159 μmol mol^−1^, an amplification of 1.20; a 4 °C rise in needle temperature amplifies Γ* by a further 1.22; the product is 1.46, giving λ = 2.30. Table 4 gives the full envelope.

Three points follow. First, the derived envelope is λ = 1.45–2.85 with a midpoint of 2.05, and the value adopted here, λ = 2.5, lies inside it. Second, 2.5 corresponds to a needle warming of roughly 4.5–5 °C at air temperatures of 28–32 °C, which is the appropriate evaluation point because the coupling term is active only when *f*_ST_ > 0, that is, during the summer stress window when closure and needle warming are both at their seasonal maximum, and not at annual-mean conditions. Third, the result is not sensitive to the choice: λ ranks eighth of ten parameters in the sensitivity analysis of Section 5.4, and setting λ = 0, which removes the coupling entirely, lowers the weighted NPP reduction from 37.9% to 31.6% and the forgone CO_2_ from 64.3 to 53.5 Mt yr^−1^. The coupling is therefore material, but no conclusion drawn here turns on whether λ is 1.5 or 2.9.

### 4.5. Biophysical Parameterization

APAR values (1000–1250 MJ m^−2^ yr^−1^) are derived from incoming PAR (≈2200–2600 MJ m^−2^ yr^−1^) and fAPAR (0.45–0.55) appropriate to closed Türkiye pine canopies; potential RUE values (0.95–1.30 g C MJ^−1^) are anchored on the MOD17 land-cover-specific RUE for evergreen needleleaf forest (0.962 g C MJ^−1^; [28]), adjusted upward to reflect the upper performance envelope of Mediterranean pines under unstressed conditions. CUE for Mediterranean conifer ecosystems is set at 0.45 (range 0.40–0.50) consistent with published values [16,20]. Wood density is 0.55 Mg m^−3^ for *P. brutia* and 0.58 Mg m^−3^ for *P. nigra*; stem allocation is 0.55 of total NPP [29,30]. Table 5 reports every parameter with its central value, range, distributional form, and source; Table 6 reports the resulting zone-level output.

The modelled actual-NPP range across the twelve units is 230–488 g C m^−2^ yr^−1^, with an area-weighted mean of 346 g C m^−2^ yr^−1^. Two independent Turkish reference points are available from the MOD17A3 evaluation of Gulbeyaz et al. [33]. The first is the country-wide MOD17A3 mean over their forest plots, 0.412 kg C m^−2^ yr^−1^ with a standard deviation equivalent to 0.23 kg C m^−2^ yr^−1^, that is 182–642 g C m^−2^ yr^−1^. The second is the forest-attributed component of the same product resolved to four regions, which ranges from 148 to 240 g C m^−2^ yr^−1^. The modelled range falls inside the first and above the second. Table 7 gives the zone-by-zone comparison against the regional values, which are the more directly comparable of the two.

Across the twelve units the modeled actual productivity exceeds the regional forest-attributed MODIS estimate by factors of 1.07 to 3.05, with an area-weighted ratio of 2.01. Three features of the comparison account for much of that gap. First, the forest-attributed quantity of Gulbeyaz et al. is obtained by partitioning the productivity of a 1 km MODIS pixel among the CORINE land-cover classes it contains, in proportion to their NDVI [33]; where a pixel is only partly forested the forest share is correspondingly diluted, so the quantity is not a per-hectare productivity of closed forest. Second, their plots follow the ICP Forests Level-1 16 km grid and therefore sample the entire national forest estate, including open and low-productivity stands, whereas the zones modelled here are closed, commercially managed pine stands selected for their production role. Third, their field-based estimates, 126 and 180 g C m^−2^ yr^−1^ by the two allometric methods, cover only the dominant trees recorded in the ministry inventory and exclude understory and non-dominant individuals, which the authors identify as a source of underestimation [33]. What none of these considerations excludes is the possibility that the potential radiation-use efficiency adopted in Section 4.5 sits at the optimistic end of its plausible range, and Section 6.4 treats that as a limitation.

The consequence is specific and worth stating precisely. The combined loss fraction of Equation (3) depends only on the stress fractions and their coupling. It is independent of absorbed PAR, of potential radiation-use efficiency, and of carbon-use efficiency, because those three quantities enter the actual and the counterfactual productivity identically and cancel in the ratio. The 37.9% reduction is therefore unaffected by the level of the baseline, and so is every proportional result reported here, including the three-tier decomposition and the drought-attributable share. The absolute deficits are not. Rescaling the baseline to the area-weighted regional MODIS value would lower the forgone sequestration from 64.3 to approximately 32 Mt CO_2_ yr^−1^ and the forgone stemwood-volume equivalent from 34.4 to approximately 17 Mm^3^ yr^−1^. The proportional findings should therefore be read as the robust result of this study and the absolute tonnages and volumes as conditional on the baseline.

### 4.6. Conversion to Stemwood and Merchantable Volume

Forgone assimilation is converted to a volume by applying the stem allocation coefficient, converting to dry mass at a carbon fraction of 0.50, and dividing by species wood density:

ΔVol_z_ = ΔNPP_z_ × *a*_stem_/*c*_frac_/ρ_z_ (5)

The quantity this yields is the volume that the unassimilated carbon would occupy if allocated to stem at the observed allocation ratio. It is a stemwood-volume equivalent, not a merchantable yield, and it is gross of mortality, self-thinning, branch shedding, stem form, top and limb deductions, rotation-length constraints, and utilization standards. We use the term “forgone stemwood-volume equivalent” throughout in place of “forgone timber volume” for this reason.

For the economic module a realization coefficient φ_R_ carries this quantity to a merchantable-equivalent one. We declare φ_R_ openly as a scoping parameter rather than a measured value: deriving it properly would require a yield-table and utilization study beyond the scope of this paper. A central value of 0.60 is adopted, with a range of 0.45–0.75 propagated through the uncertainty analysis, and results are reported on both the gross and the merchantable basis throughout Section 5.

### 4.7. Uncertainty Propagation

Two complementary procedures are used. The first is a one-at-a-time sensitivity analysis, in which each parameter is moved to the low and high end of its range while all others are held at their central values, and the resulting change in the annual deficit is recorded; this isolates the marginal influence of each input and is reported as the tornado diagram presented in Section 5.4. The parameters varied, and their ranges are those listed in Table 5. The one-at-a-time procedure cannot capture interactions or joint probability, and it is therefore reported alongside, and not in place of, the Monte Carlo analysis.

The second procedure is a Monte Carlo simulation with N = 40,000 draws. Parameters are sampled independently from the distributions given in Table 5 on each draw, the full zone-level model is evaluated, and the national aggregates are recorded, yielding an empirical distribution for every reported quantity. Two features of the sampling design deserve statement. The stress fractions are drawn with 70% of their variance shared across zones and 30% being zone-independent, because they derive from a common literature base and are therefore correlated; treating them as independent would understate the aggregate uncertainty through spurious cancellation. The carbon price is drawn from a right-skewed triangular distribution whose mode is USD 30 t^−1^ but whose mean is USD 41.7 t^−1^, which is why the Monte Carlo medians for the economic quantities in Section 5.5 exceed the central estimates computed at modal prices.

### 4.8. Economic Translation and Discounting

Forgone stemwood volume is valued at a central pine-stumpage price of USD 70 m^−3^, with sensitivity bounds of USD 55 m^−3^ (low) and USD 90 m^−3^ (high), reflecting the observed range of OGM pine-sawlog auction prices over 2020–2023 [2]. Forgone CO_2_ (=3.67 × forgone NPP-C) is valued at central USD 30 t^−1^ CO_2_, with low USD 15 and high USD 80 bounds spanning the voluntary-market mean and the EU-ETS Phase 4 envelope, respectively [31,32]. Time-series valuation over 2000–2023 incorporates the following: (i) a 0.6% yr^−1^ area growth factor reflecting observed OGM-reported forest-area expansion; (ii) a 1.2% yr^−1^ real timber-price growth; and (iii) a linear ramp of the carbon price from USD 5 t^−1^ in 2000 to USD 30 t^−1^ in 2023.

Net present values are computed at a 4% real discount rate to a 2023 base year. The convention applied is that each annual flow in year *t* is weighted by (1 + *r*)^−(2023−t)^, so that the terminal year carries unit weight and earlier years are discounted progressively; the convention is stated explicitly so that the reported present value can be reproduced.

## 5. Results

### 5.1. Decomposition of the Weighted-Mean Carbon Flow into Obligate and Drought-Attributable Losses

**Figure 3:**
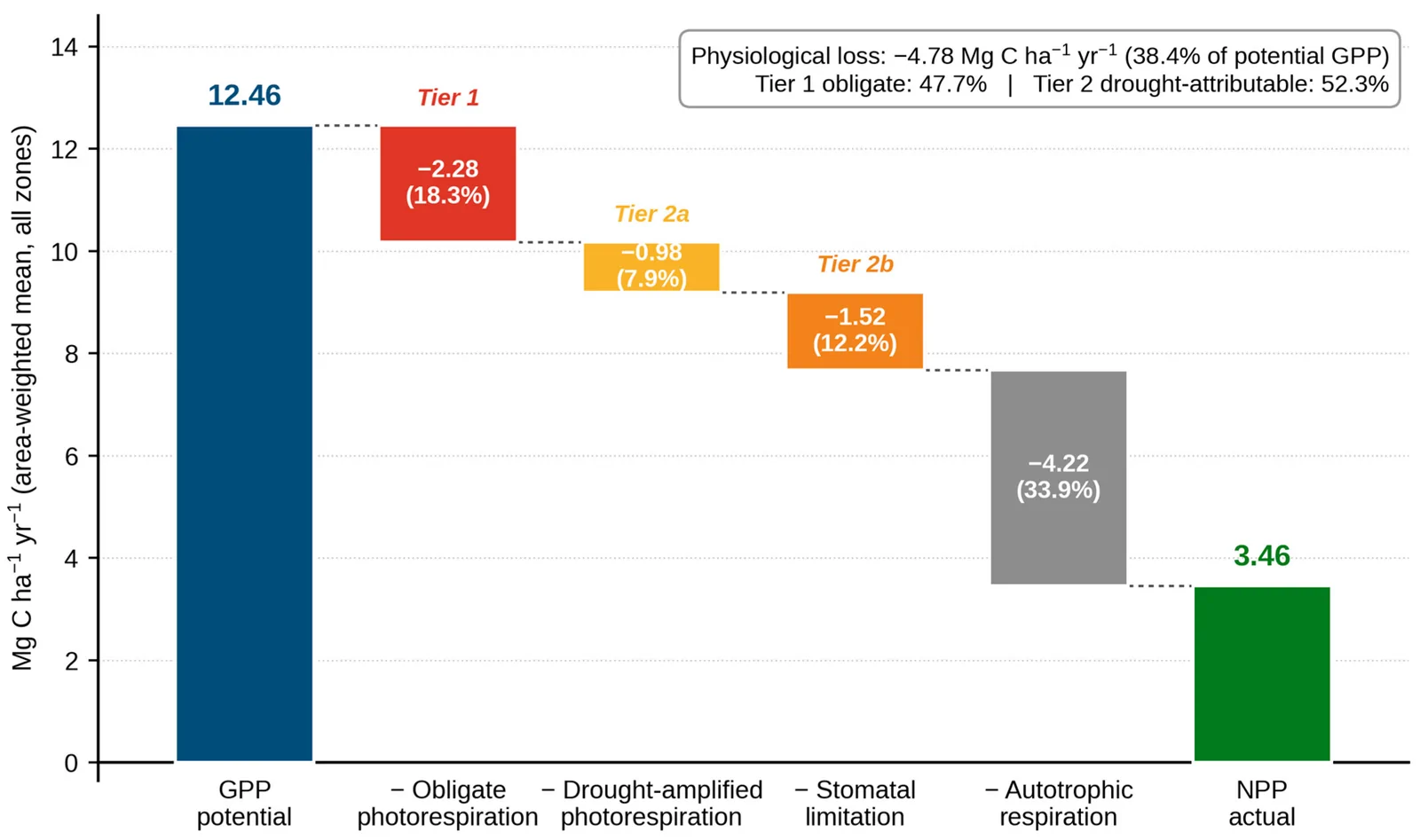
Waterfall decomposition of the weighted-mean carbon flow from potential GPP to actual NPP across the combined *P. brutia* and *P. nigra* range in Türkiye. Photorespiration and stomatal limitation jointly remove 4.78 Mg C ha^−1^ yr^−1^, or 38.4% of potential GPP. The photorespiration term is separated into the obligate metabolic component (Tier 1), which is not addressable by management, and the drought-amplified increment (Tier 2a), which is. Autotrophic respiration is shown for completeness and is not part of the physiological deficit.

Weighting each zone × species combination by its standing area, the potential GPP for the combined 8.15 Mha range comes to 12.46 Mg C ha^−1^ yr^−1^, and actual NPP to 3.46 Mg C ha^−1^ yr^−1^. The physiological loss separating them is 4.78 Mg C ha^−1^ yr^−1^, or 38.4% of potential GPP, and it resolves into three components of very different management status (Figure 3, Table 8).

Tier 1 is the obligate photorespiratory cost, the value the fraction takes under well-watered conditions at each zone’s thermal regime. It is the metabolic floor of C_3_ carboxylation, and no silvicultural intervention addresses it. Tier 2a is the drought amplification of that same photorespiratory term, arising through the diffusive and thermal routes of Section 4.4. Tier 2b is stomatal limitation itself. Only Tier 2 is even in principle amenable to management, and it accounts for slightly more than half of the loss: 20.1 of the 37.9 percentage points by which potential NPP is reduced, and 33.6 of the 64.3 Mt CO_2_ yr^−1^ reported in Section 5.2.

Autotrophic respiration accounts for the largest single step in Figure 3, but it is a standard metabolic cost of tissue construction and maintenance rather than a stress-induced loss, and it forms no part of the deficit quantified here; it is shown only so that the path from potential GPP to realized NPP is complete. The same distinction operates within the photorespiration term itself, which Figure 3 separates into its obligate component and its drought-amplified increment.

### 5.2. Aggregate Stemwood and Carbon Deficits

Scaling the zonal output across the full 8.15 Mha range yields an annual forgone CO_2_ sequestration of 64.3 Mt CO_2_ yr^−1^ and a forgone stemwood-volume equivalent of 34.4 Mm^3^ yr^−1^ (Table 9). Applying the realization coefficient of Section 4.6 gives a merchantable-equivalent volume of 20.6 Mm^3^ yr^−1^, with a range of 15.5–25.8 Mm^3^ yr^−1^ across the φ_R_ bounds. For context, Turkey’s entire 2022 harvest came to 25 Mm^3^: the physiological deficit is therefore of the same order as the national annual cut from all species combined, rather than larger than it as the gross figure alone would suggest. This is timber that does not exist in the bole, because the carbon was never assimilated.

### 5.3. Cumulative and Present-Value Deficit over the 2000–2023 Reference Period

**Figure 4:**
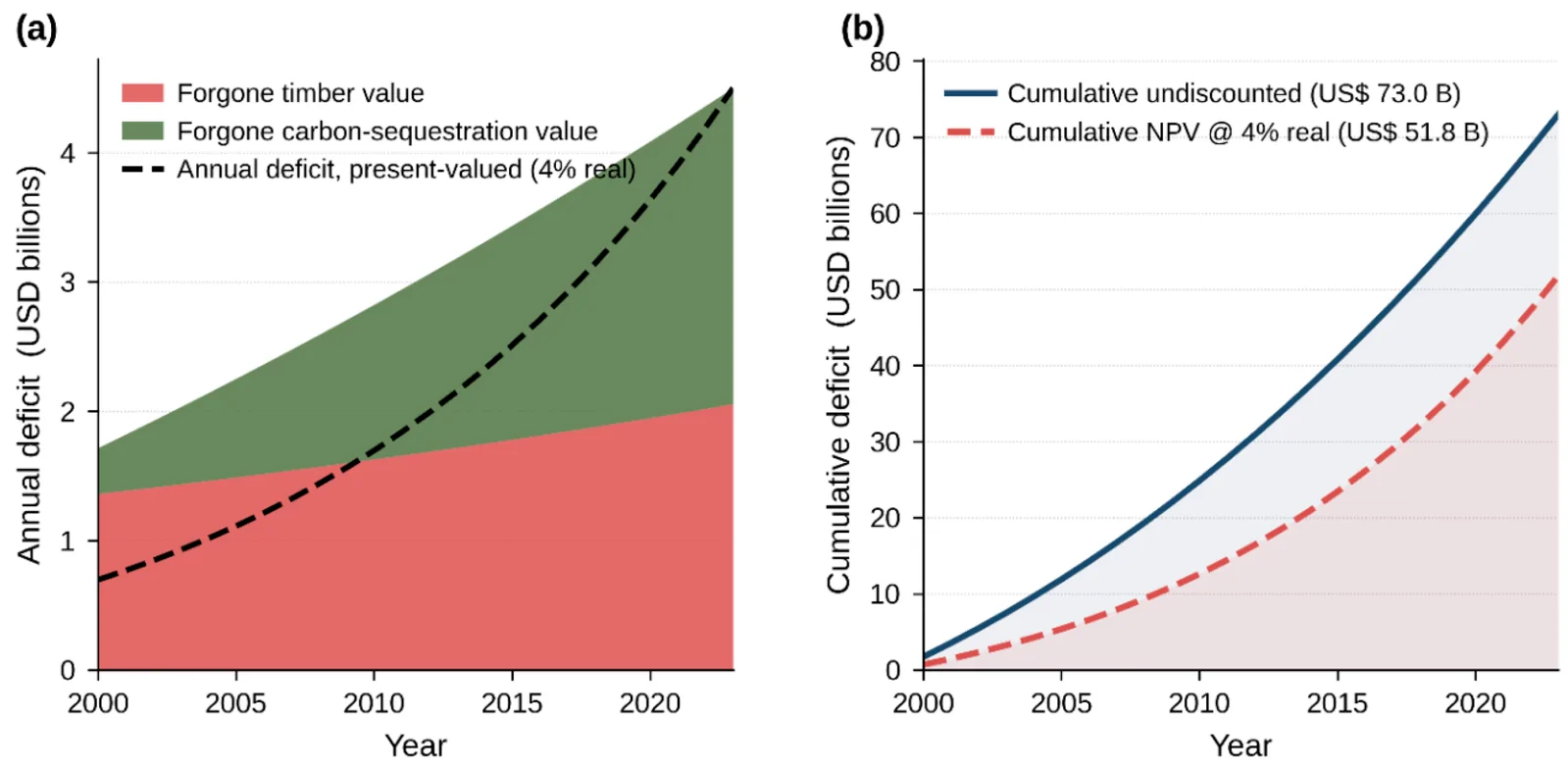
(**a**) Annual economic deficit attributable to photorespiration and drought stress in Türkiye’s *P. brutia* and *P. nigra* forests, decomposed into forgone-timber and forgone-carbon components, and the present-value annual deficit (4% real discount rate). (**b**) Cumulative deficit, 2000–2023, undiscounted vs. NPV at 4% real. Both panels are computed on the gross stemwood basis; the merchantable-basis equivalents are given in Table 9.

When the annual deficit is propagated across the 24-year reference period, with area growth at 0.6% yr^−1^, real timber-price growth at 1.2% yr^−1^, and the carbon-price ramp described in Section 4.8, the undiscounted cumulative deficit reaches approximately USD 73 billion on the gross basis. Applying a 4% real discount rate to 2023 dollars gives a net present value close to USD 51 billion (Figure 4). This number should not be read as a realized monetary loss: the counterfactual world is unachievable without altering plant biochemistry itself. It should be read instead as a measure of the economic stakes the two physiological constraints impose on Türkiye’s pine sector.

### 5.4. Sensitivity of the Annual Economic Deficit to Model Parameters

**Figure 5:**
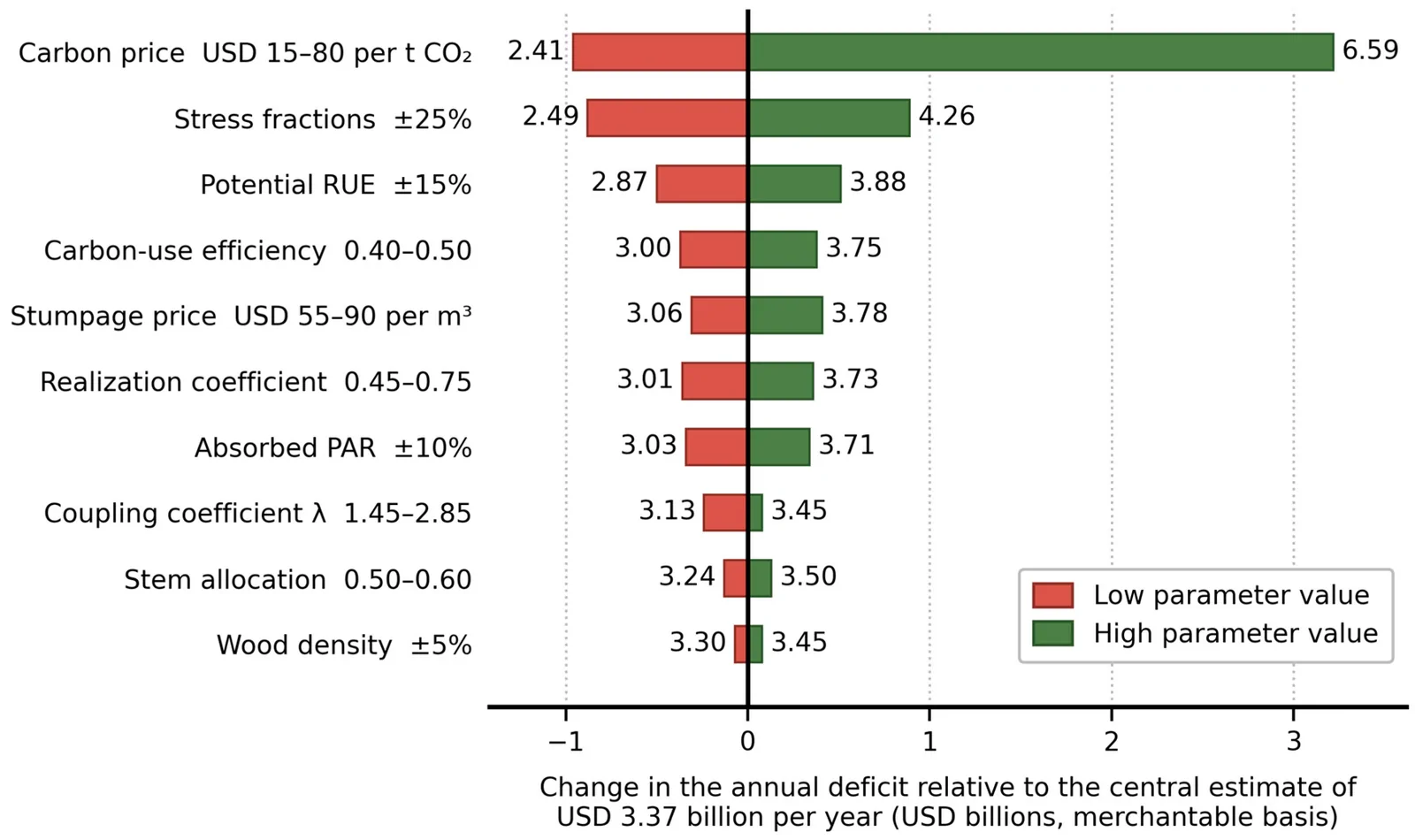
Tornado sensitivity of the annual economic deficit around the central estimate of USD 3.37 B yr^−1^ on the merchantable basis. Each parameter is moved to the low and high end of the range given in Table 5 while all others are held at their central values. Carbon price exerts the largest single influence; the qualitative result is robust across all tested parameter ranges.

A one-at-a-time tornado analysis around the central USD 3.37 B yr^−1^ merchantable-basis estimate (Figure 5) indicates that carbon price is by a wide margin the single most influential input, followed by the magnitude of the stress fractions and by potential RUE. The coupling coefficient λ ranks eighth of the ten parameters tested. Even at the simultaneous pessimistic bound of all ten parameters—stress fractions −25%, RUE −15%, APAR −10%, CUE = 0.40, λ = 1.45, stem allocation 0.50, wood density +5%, φ_R_ = 0.45, stumpage USD 55 m^−3^ and carbon USD 15 t^−1^ CO_2_—the annual deficit remains USD 0.81 billion yr^−1^ on the merchantable basis, the forgone sequestration remains 30.5 Mt CO_2_ yr^−1^, and the weighted NPP reduction remains 26.5%. The physical magnitude of the constraint is therefore robust across the parameter space even where its monetary expression is not.

The parameter that matters most for the policy inference, rather than for the magnitude of the estimate, is the carbon price, and its influence is discontinuous in character rather than merely large. On the merchantable-equivalent basis, the forgone-carbon component overtakes the forgone-timber component at a carbon price of approximately USD 22 t^−1^ CO_2_. Below that threshold the physiological deficit reads as a forest-products problem, and the appropriate policy response lies in silvicultural and yield regulation; above it the deficit reads primarily as a climate-accounting problem, and the appropriate response shifts towards inventory design, baseline specification, and mitigation policy. Türkiye entered this range during the reference period, and both the EU ETS envelope and the emerging domestic pricing framework sit above it [31,34]. The framing of the result, and not only its size, therefore depends on where the carbon price settles.

### 5.5. Uncertainty Propagation and Confidence Intervals

Monte Carlo propagation of the parameter distributions in Table 5 through the full zone-level model gives the confidence intervals reported in Table 10. The biophysical medians sit marginally below the central estimates because the stress-fraction multiplier is symmetric, while the model response to it is mildly concave; the economic medians sit above them because the carbon-price distribution is right-skewed, as noted in Section 4.7.

Two robustness statements follow. The annual deficit exceeds USD 3 billion in 98.8% of draws, and the forgone sequestration exceeds 40 Mt CO_2_ yr^−1^ in 99.9% of draws. Ranking the drivers by Spearman rank correlation with the annual deficit gives carbon price (0.76), absorbed PAR (0.33), the stress fractions (0.32), potential RUE (0.26), stumpage price (0.20), carbon-use efficiency (0.19), the coupling coefficient λ (0.09) and stem allocation (0.08). The qualitative conclusion—that the eco-physiological discount on these two species is large, is dominated on the stomatal side in the continental interior, and is only about half addressable by management—holds across the entire sampled parameter space.

## 6. Discussion

### 6.1. Silvicultural Leverage on the Drought-Attributable Component

The decomposition in Section 5.1 fixes the ceiling on what silviculture can do. Tier 1 is unavailable at any intensity of intervention, so the entire management question concerns the 20.1 percentage points of Tier 2, and within Tier 2 the stomatal term dominates in inner Anatolia and the semi-arid transition. Three responses act on that term.

The first is thinning. In precommercially thinned stands of *P. nigra* subsp. *pallasiana* in the western Mediterranean and semi-arid interior of Türkiye, soil water content, midday xylem water potential, net assimilation rate, and stomatal conductance were all higher than in unthinned controls, and the assimilation response tracked soil water content and midday water potential directly [35,36]. Needle chlorophyll concentration moved in the opposite direction, declining after thinning [35,36], which indicates that the assimilation gain is driven by improved water status and light environment rather than by pigment-level acclimation. The wider meta-analytic evidence supports the same mechanism across conifers, with benefits scaling with thinning intensity and decaying as the canopy recloses [37]. Because the mechanism addressed is precisely the Tier 2b term that accounts for 31.7% of the physiological loss, thinning acts directly on the largest single management-addressable component identified here. Initial spacing exerts the same lever at establishment in *P. brutia* plantations, where it measurably alters early growth rate and carbon sequestration [38].

The second response is portfolio mixing. *P. brutia* and *P. nigra* have complementary stress vulnerabilities: *P. brutia* carries the higher photorespiratory risk at low elevations but tolerates summer drought better; *P. nigra* is more drought-sensitive at its lower margin but more cold-tolerant. The 800–1200 m elevational belt in which the two species naturally meet therefore offers a diversification at the stand scale, smoothing the summer carbon deficit across the landscape [19,26]. The third is provenance selection and assisted migration: *P. brutia* is known to carry roughly 150 chloroplast haplotypes [7], with measurably higher drought tolerance in dryland-origin populations and a productive interspecific hybrid (*P. brutia* × *P. halepensis*) that combines drought-tolerance traits from each parent [7,15,39].

How much of the deficit these interventions could actually recover is a separate question and one the present evidence base cannot answer precisely. The Turkish thinning literature establishes the direction and the mechanism of the response but does not support a stand-to-landscape scaling of the carbon response over a full drought cycle. We therefore report scenarios over a stated recovery efficiency η rather than a single estimate (Table 11). At η = 0.20, silviculture addresses approximately four percentage points of a thirty-eight point reduction. That is a real and policy-relevant quantity, and it is an order of magnitude smaller than the counterfactual gap—which is exactly why the two must not be reported as though they were interchangeable.

### 6.2. Projected Intensification of Both Constraints Under CMIP6 Scenarios

The losses computed here are anything but static. Under CMIP6 ensembles for the eastern Mediterranean, summer temperatures over Türkiye are projected to increase by 2.5–6.5 °C, and summer precipitation is projected to decrease by 10–40% by 2100 relative to the 1981–2010 baseline, with the upper bounds concentrated in Central Anatolia and the Lakes District [40,41,42]. Both shifts amplify our two stressors: photorespiration rises because the RuBisCO specificity factor S*c/o* falls with temperature [21,22,43], and stomatal limitation worsens because soil moisture declines while VPD increases [44,45]. Process-based simulations of *P. brutia* under RCP4.5 and RCP8.5 produce declining NEP trajectories for both 2051–2060 and 2091–2100 horizons relative to the 1980–2020 baseline [16].

There is a tension between those trajectories and our own parameterization that is worth making explicit rather than leaving to the reader. Our stress fractions are held at present-day values throughout the reference period, whereas the projections imply that both rise. Our estimate of the physiological discount is therefore conservative by construction and conservative in a direction that can be signed. Applying the upper end of the ±25% plausibility interval—a rough proxy for the fractions a warmer, drier late-century climate would imply—raises the weighted NPP reduction from 37.9% to 43.4% and the annual deficit from USD 3.37 to USD 4.26 billion on the merchantable basis. The direction of travel is unambiguous even if the magnitude is not: the stakes this counterfactual identifies grow rather than shrink, and they grow fastest in exactly the zones where the stomatal term already dominates.

### 6.3. From Sequestration Deficit to Creditable Mitigation Outcome

A sequestration deficit is not a credit supply. The 64.3 Mt CO_2_ yr^−1^ identified in Section 5.2 is a shadow quantity: it measures carbon that the two species did not assimilate, and it is properly compared with national inventory totals and with the social cost of carbon. It is not a quantity that could be issued into a compliance or voluntary market, and the distinction is not a technicality. Crediting requires that a ton be additional to the baseline, that the baseline itself be credibly specified, that the activity not simply displace emissions elsewhere, and that the storage be durable. Each requirement removes a further portion of the deficit from eligibility (Table 12).

The cascade yields approximately 1.0 Mt CO_2_ yr^−1^ of potentially issuable credits, some 1.6% of the headline deficit—USD 0.03 billion yr^−1^ at USD30 t^−1^, against USD 1.93 billion if the deficit were monetized directly. The two quantities differ by a factor of roughly sixty, and reporting the larger one as though it were the smaller is the error this section is designed to prevent.

Two features of the Turkish case make even the smaller figure fragile. Baseline specification is unusually demanding here because the counterfactual is itself declining: the trajectories reviewed in Section 6.2 imply that both stress fractions rise over any plausible crediting period, so a static baseline would credit as additional a stand that is merely declining more slowly than its neighbors. Dynamic baselines are therefore a requirement rather than a refinement in this system. Additionally, it is separately constrained by the fact that thinning is already a programmed activity within OGM stand tending, which removes a substantial share of any intervention from the additional category by construction. The experience of forest-offset programs elsewhere is directly relevant: a systematic evaluation of California’s compliance forest offsets found net over-crediting of roughly 29% of the credits analyzed, arising principally from baseline design rather than from measurement error [46]. Türkiye is building its domestic carbon-pricing framework alongside its net-zero commitment for 2053 [34], and the Article 6 mechanisms of the Paris Agreement provide an institutional vehicle [31,47]; the empirical case made here is for careful baseline design within that vehicle, not for treating the physiological deficit as an available credit stream.

### 6.4. Structural Limitations of the Counterfactual Framework

Eight limitations merit explicit statement. (i) Potential GPP under the counterfactual is computed from an RUE × APAR framework anchored on MOD17 values [28,33]; the truly unconstrained optimum cannot be directly observed, and the framework therefore carries inherent uncertainty. (ii) The stress fractions of Table 3 are a structured elicitation bounded by published process constraints, not a statistical estimation; the paired gas-exchange and flux campaign that would replace elicitation with measurement is the single most valuable next step and would require thinned and unthinned plots instrumented across a full drought cycle in at least the Mediterranean Coast, Central Anatolia, and Aegean Hinterland zones. (iii) Turkey’s pine forests exhibit large within-zone variability in soils, microclimate, and stand history, which zone-level aggregation smooth over. (iv) The coupling term of Section 4.4 is a first-order approximation whose derivation assumes that needle warming scales linearly with the degree of closure; the true relationship becomes increasingly non-linear at the extreme end of combined stress. (v) The realization coefficient φ_R_ is a scoping parameter and not a measured quantity, and the merchantable-equivalent volumes should be read accordingly. (vi) Carbon prices between 2000 and 2023 ranged from near zero in early voluntary markets to above EUR 80 t^−1^ in EU ETS Phase 4 [31,32]; the USD 15–80 envelope is broad but cannot capture all possible policy trajectories. (vii) Species-area data at sub-regional resolution remain limited; access to OGM Regional Directorate inventories would further refine these estimates. (viii) The level of the baseline is the largest single uncertainty in the absolute results. As Section 4.5 shows, the modeled productivity is about twice the forest-attributed MODIS estimate of Gulbeyaz et al. [33] across the twelve zones. Part of that gap is explained by the way that quantity is constructed and by the different populations of stands sampled, but part of it may reflect a potential radiation-use efficiency set too high. Because the loss fraction is a ratio in which absorbed PAR, radiation-use efficiency, and carbon-use efficiency cancel, the proportional results are unaffected; the tonnages and volumes would scale approximately in proportion to any revision of the baseline and could be as much as halved.

### 6.5. What This Study Adds

Three things follow from this analysis that did not follow from the constituent literatures separately. The first is a magnitude: the eco-physiological discount on Türkiye’s two dominant conifers is of the order of a third of potential productivity, which places it among the larger single terms in the national forest carbon balance and above the level at which it can reasonably be absorbed into model residuals. The second is a partition: slightly more than half of that discount is drought-attributable and therefore in principle responsive to management, while slightly less than half is the obligate metabolic cost of C_3_ carboxylation and is responsive to nothing available to a forest manager. Conflating the two, as the forest-management literature has generally conducted when invoking physiological limitation, systematically overstates what silviculture can deliver. The third is a market implication: the gap between a sequestration deficit and an issuable credit is not a detail of accounting practice but a factor of roughly sixty in this system, and it widens rather than narrows under a declining baseline. Any offset framework built on Türkiye’s conifer estate that does not use dynamic baselines will over-credit, and the size of the physiological discount identified here indicates by how much.

## 7. Conclusions

Photorespiration and drought-induced stomatal limitation jointly suppress the productivity of Türkiye’s two dominant commercial conifers by 37.9% of radiation-limited potential (95% CI 32.2–43.4%) across an estate of 8.15 Mha. The suppression corresponds to 64.3 Mt CO_2_ yr^−1^ of forgone sequestration (47.8–81.2) and to 34.4 Mm^3^ yr^−1^ of forgone stemwood-volume equivalent (24.9–44.5), of which approximately 20.6 Mm^3^ would be merchantable.

That deficit is not a single quantity. Slightly less than half of it is the obligate metabolic cost of C_3_ carboxylation, which no silvicultural intervention can address. The drought-attributable remainder—20.1 of the 37.9 percentage points—is the only portion on which management acts, and at a plausible recovery efficiency the realistic gain is of the order of four percentage points. The counterfactual is therefore an accounting benchmark for the physiological discount, not a target for forest management, and the two should not be reported as though they were interchangeable.

The same distinction governs the market implication. Valued as a shadow quantity the deficit is substantial, but filtered for eligible area, recovery efficiency, additionality, leakage, and permanence, it yields approximately 1.0 Mt CO_2_ yr^−1^ of issuable credits, under two per cent of the headline figure. Because the baseline itself declines under every CMIP6 trajectory examined, static baselines applied to this estate would systematically over-credit.

The wider implication is methodological. Eco-physiological suppression of this magnitude is currently invisible in national forest carbon accounting, which measures what stands do rather than what they are constrained not to do. Making it visible changes the interpretation of sink estimates, the design of dynamic baselines, and the credibility of offsets generated from Mediterranean conifer forests.

## Figures and Tables

**Table 1:** Regional distribution and productivity parameters of *Pinus brutia* in Türkiye.

Zone	Area (10^3^ ha)	Elevation (m)	MAP (mm)	MAI (m^3^ ha^−1^ yr^−1^)	Primary Use
Mediterranean Coast (0–500 m)	1500	0–500	600–1200	3.5–6.2	Timber, resin
Mediterranean Mid (500–1000 m)	1200	500–1000	600–1100	3.0–5.5	Timber, watershed
Aegean	700	0–900	500–900	2.8–5.0	Timber, afforestation
Marmara	250	0–600	600–900	2.5–4.5	Timber, urban green
Interior Transitional	250	500–1200	300–600	1.8–3.0	Erosion control
Black Sea (isolated stands)	50	0–500	800–1400	2.0–3.5	Watershed protection

Sources: [1,2,5,7,19].

**Table 2:** Regional distribution and productivity parameters of *Pinus nigra* subsp. *pallasiana* in Türkiye.

Zone	Area (10^3^ ha)	Elevation (m)	MAP (mm)	MAI (m^3^ ha^−1^ yr^−1^)	Primary Use
Central Anatolia	1200	800–1800	300–500	1.5–3.0	Timber, erosion
Mediterranean Mts. (Taurus)	1000	800–2000	500–900	2.5–4.5	Timber, watershed
Aegean Hinterland	700	500–1600	400–700	2.0–3.8	Timber, afforestation
Marmara/NW Anatolia	500	300–1400	500–800	2.2–4.0	Industrial timber
Eastern Anatolia	400	1000–2000	350–600	1.2–2.5	Erosion control
Black Sea Transition	400	500–1800	600–1000	2.5–4.2	Timber, watershed

Sources: [1,2,4,8,18].

**Table 3:** Estimated proportional GPP reduction attributable to photorespiration and drought-induced stomatal limitation, by zone and species. Plausibility intervals are ±25% of the central value in each case.

Zone	Species	Photoresp. Loss *f*_PR_ (% GPP)	Stomatal Loss *f*_ST_ (% GPP)	Combined NPP Reduction (%)	Evidence
Mediterranean Coast (0–500 m)	*P. brutia*	25 (19–31)	20 (15–25)	50	[15,16]
Mediterranean Mid (500–1000 m)	*P. brutia*	18 (14–23)	14 (11–18)	35	[16,19]
Aegean	*P. brutia*	20 (15–25)	16 (12–20)	40	[15]
Marmara	*P. brutia*	13 (10–16)	11 (8–14)	26	[16]
Interior Transitional	*P. brutia*	20 (15–25)	20 (15–25)	44	[18,19]
Black Sea (isolated stands)	*P. brutia*	10 (8–13)	7 (5–9)	18	[16]
Central Anatolia	*P. nigra*	15 (11–19)	25 (19–31)	43	[18]
Mediterranean Mts. (Taurus)	*P. nigra*	16 (12–20)	14 (11–18)	33	[14]
Aegean Hinterland	*P. nigra*	18 (14–23)	17 (13–21)	38	[18]
Marmara/NW Anatolia	*P. nigra*	13 (10–16)	11 (8–14)	26	[16]
Eastern Anatolia	*P. nigra*	12 (9–15)	22 (17–28)	37	[14]
Black Sea Transition	*P. nigra*	10 (8–13)	7 (5–9)	18	[16]

Combined NPP reduction = 1 − [1 − *f*_PR_(1 + λ *f*_ST_)](1 − *f*_ST_), with λ = 2.5 (Section 4.4). Two rows absent from the submitted version—*P. nigra* · Marmara/NW Anatolia, and the separation of the Black Sea Transition row into its two species—have been added so that Table 3 corresponds one-to-one with the twelve modelled zone × species units of the counterfactual model (Section 4.5).

**Table 4:** Derivation of the coupling coefficient λ, evaluated at *f*_ST_ = 0.20. Reference condition: *C*ₐ = 400 μmol mol^−1^, *C*_i_/*C*ₐ = 0.65, *A* = 10 μmol m^−2^ s^−1^. Rubisco kinetics after [9]. The complete derivation is given as Supplementary Text S1.

Evaluation Condition	Diffusive Amplification	Thermal Amplification	Combined	λ
28 °C, Δ*T*_leaf_ 4 °C	1.198	1.219	1.461	2.30
32 °C, Δ*T*_leaf_ 4 °C	1.173	1.213	1.423	2.12
36 °C, Δ*T*_leaf_ 4 °C	1.149	1.207	1.387	1.94
28 °C, Δ*T*_leaf_ 5 °C	1.198	1.278	1.531	2.67
Diffusive route only (Δ*T*_leaf_ = 0)	1.15–1.20	1.000	1.15–1.20	0.75–0.99
Envelope, *T* 28–36 °C, Δ*T*_leaf_ 3–5 °C	—	—	—	1.45–2.85

**Table 5:** Model parameters: central values, ranges, distributional forms used in the uncertainty analysis, and sources.

Parameter	Central	Range	Distribution	Source
APAR (MJ m^−2^ yr^−1^)	850–1250 by zone	±10%	Truncated normal	Derived from PAR and fAPAR
RUE_pot_ (g C MJ^−1^)	0.95–1.30 by zone	±15%	Triangular	[28]
CUE	0.45	0.40–0.50	Triangular	[16,20]
*f*_PR_, *f*_ST_	Table 3	±25%	Triangular, 70% shared	Section 4.3
λ (coupling)	2.5	1.45–2.85	Triangular	Section 4.4
Stem allocation	0.55	0.50–0.60	Triangular	[29,30]
Carbon fraction of dry mass	0.50	—	Fixed	[30]
Wood density (Mg m^−3^)	0.55/0.58	±5%	Truncated normal	[29]
Realization coefficient φ_R_	0.60	0.45–0.75	Triangular	Section 4.6, scoping parameter
Stumpage price (US$ m^−3^)	70	55–90	Triangular	[2]
Carbon price (US$ t^−1^ CO_2_)	30	15–80	Triangular	[31,32]
Discount rate (real)	4%	—	Fixed	Section 4.8

**Table 6:** Zone-level model output under the central parameter set.

Zone × Species	Area (10^3^ ha)	APAR (MJ m^−2^)	RUE_pot_	*f*_comb_	NPP_act_/NPP_cf_ (Mg C ha^−1^)	ΔVol (m^3^ ha^−1^)
*P. brutia* · Mediterranean Coast	1500	1250	1.30	0.500	3.66/7.31	7.31
*P. brutia* · Mediterranean Mid	1200	1150	1.25	0.349	4.21/6.47	4.51
*P. brutia* · Aegean	700	1100	1.20	0.395	3.59/5.94	4.69
*P. brutia* · Marmara	250	1000	1.15	0.258	3.85/5.18	2.67
*P. brutia* · Interior Transitional	250	950	1.10	0.440	2.63/4.70	4.14
*P. brutia* · Black Sea	50	1050	1.15	0.179	4.46/5.43	1.95
*P. nigra* · Central Anatolia	1200	900	1.00	0.433	2.30/4.05	3.32
*P. nigra* · Mediterranean Mts.	1000	1050	1.15	0.326	3.66/5.43	3.36
*P. nigra* · Aegean Hinterland	700	1000	1.10	0.383	3.05/4.95	3.59
*P. nigra* · Marmara/NW	500	1000	1.10	0.258	3.68/4.95	2.42
*P. nigra* · Eastern Anatolia	400	850	0.95	0.365	2.31/3.63	2.52
*P. nigra* · Black Sea Transition	400	1100	1.20	0.179	4.88/5.94	2.02

**Table 7:** Zone-level modelled net primary productivity against the forest-attributed MODIS estimates of Gulbeyaz et al. [33]. All productivity values are g C m^−2^ yr^−1^; the ratio is modeled over MODIS.

Zone × Species	Modelled NPP_act_	Modelled NPP_cf_	Gulbeyaz et al. [33] Region	MODIS Forest NPP	Ratio
*P. brutia* · Mediterranean Coast	366	731	Aegean and Mediterranean	148	2.47
*P. brutia* · Mediterranean Mid	421	647	Aegean and Mediterranean	148	2.84
*P. brutia* · Aegean	359	594	Aegean and Mediterranean	148	2.43
*P. brutia* · Marmara	384	518	W. Black Sea and Marmara	240	1.60
*P. brutia* · Interior Transitional	263	470	Central and E. Anatolia	214	1.23
*P. brutia* · Black Sea	446	543	Mid and E. Black Sea	160	2.79
*P. nigra* · Central Anatolia	230	405	Central and E. Anatolia	214	1.07
*P. nigra* · Mediterranean Mts.	366	543	Aegean and Mediterranean	148	2.47
*P. nigra* · Aegean Hinterland	305	495	Aegean and Mediterranean	148	2.06
*P. nigra* · Marmara/NW	368	495	W. Black Sea and Marmara	240	1.53
*P. nigra* · Eastern Anatolia	231	363	Central and E. Anatolia	214	1.08
*P. nigra* · Black Sea Transition	488	594	Mid and E. Black Sea	160	3.05

**Table 8:** Three-tier decomposition of the weighted-mean physiological carbon loss.

Component	Mg C ha^−1^ yr^−1^	% of Potential GPP	% of the Loss	Mt CO_2_ yr^−1^
Tier 1—obligate photorespiration	2.28	18.3	47.7	30.6
Tier 2a—drought-amplified photorespiration	0.98	7.9	20.6	13.2
Tier 2b—stomatal limitation	1.52	12.2	31.7	20.4
Total physiological loss	4.78	38.4	100.0	64.3
Tier 2 combined (management-addressable)	2.50	20.1	52.3	33.6

**Table 9:** Aggregate annual deficit, with central estimates, on both the gross stemwood and merchantable bases.

Quantity	*P. brutia*	*P. nigra*	Combined
Total area (10^3^ ha)	3950	4200	8150
Annual ΔNPP (10^6^ Mg C yr^−1^)	10.7	6.8	17.5
Forgone CO_2_ (10^6^ Mg CO_2_ yr^−1^)	39.4	24.9	64.3
Forgone stemwood equivalent (10^6^ m^3^ yr^−1^)	21.5	12.9	34.4
Merchantable equivalent at φ_R_ = 0.60 (10^6^ m^3^ yr^−1^)	12.9	7.7	20.6
Forgone timber value, merchantable (USD B)	0.90	0.54	1.44
Forgone carbon shadow value (USD B)	1.18	0.75	1.93
Annual total deficit, merchantable basis (USD B)	2.08	1.29	3.37
Annual total deficit, gross basis (USD B)	2.68	1.65	4.33

Stumpage price USD 70 m^−3^; carbon price USD 30 t^−1^ CO_2_. Central estimates; full uncertainty in Table 10.

**Table 10:** Monte Carlo results, N = 40,000 draws.

Quantity	Central Estimate	Monte Carlo Median	95% Confidence Interval
Weighted NPP reduction (%)	37.9	37.7	32.2–43.4
Forgone CO_2_ (Mt CO_2_ yr^−1^)	64.3	62.8	47.8–81.2
Forgone stemwood equivalent (Mm^3^ yr^−1^)	34.4	33.6	24.9–44.5
Merchantable equivalent (Mm^3^ yr^−1^)	20.6	20.1	13.9–28.3
Drought-attributable share of loss (%)	52.3	51.7	49.4–53.1
Tier 2 deficit (Mt CO_2_ yr^−1^)	33.6	32.3	24.4–42.2
Annual deficit, merchantable basis (USD B)	3.37	3.94	2.40–6.48
Annual deficit, gross basis (USD B)	4.33	4.92	3.19–7.64
Cumulative 2000–2023, NPV at 4% (USD B)	51	57	38–86

**Table 11:** Recovery-potential scenarios, applied to the Tier 2 drought-attributable component only. These are scenarios over a stated parameter, not estimates.

Recovery Efficiency η	Percentage Points of the 37.9% Reduction	Mt CO_2_ yr^−1^ Recovered	Mm^3^ yr^−1^ Stemwood Equivalent
0.10	2.0	3.4	1.8
0.20	4.1	6.7	3.6
0.30	6.1	10.1	5.4

**Table 12:** Credit-eligibility cascade applied to the Tier 2 drought-attributable deficit. Each factor is a stated scenario value.

Filter Step	Factor	Mt CO_2_ yr^−1^	Rationale
Tier 2 drought-attributable deficit	—	33.6	Table 8
Eligible area share	0.35	11.8	Stands suitable for water-oriented silviculture
Recovery efficiency η	0.20	2.4	Section 6.1
Additionality	0.60	1.4	Net of business-as-usual OGM stand tending
Leakage	0.90	1.3	Activity-shifting and market leakage
Permanence buffer	0.80	1.0	Fire and drought-reversal buffer pool

## Data Availability

All data used in this analysis are derived from published sources cited in the references. Model code, the parameter file, and the Monte Carlo implementation used to generate Table 8, Table 9, Table 10, Table 11 and Table 12 and Figure 3 and Figure 5 are available from the corresponding author on reasonable request.

## Supplementary Materials

The following supporting information can be downloaded at https://www.mdpi.com/article/10.3390/plants15162527/s1: Table S1: Derivation audit trail for the zone-specific stress fractions of Table 3, giving for each zone × species unit the evidence invoked, what that evidence establishes, the transformation applied, and the fraction adopted; Table S2: Diagnostic consistency check of the adopted fractions against a mechanical mapping from elevation and precipitation; Table S3: Derivation of λ at *f*_ST_ = 0.20 under the reference condition of Section S1.4; Text S1: Complete derivation of the coupling coefficient λ from Rubisco kinetics and leaf energy balance, with Table S3 giving the derived envelope; Figure S1: Sensitivity of λ to air temperature, to the stomatal loss fraction, and to the assumed needle-warming term.
