# Supplementary material for: Quantifying Carbon Losses Associated with Photorespiration and Drought Stress in Two Dominant Mediterranean Pine Species
Source: Plants (Basel). 2026 Aug 20;15(16):2527. doi: 10.3390/plants15162527 (PMC13516784; doi:10.3390/plants15162527)
Supplement: Supplementary file 1 [file plants-15-02527-s001.zip › plants-4483351-supplementary.pdf]

Supplementary Materials

Quantifying Carbon Losses Associated with Photorespiration and Drought Stress in Two Dominant Mediterranean Pine Species

Emre Yazar, Bülent Akgün and Emre Babur

Manuscript ID: plants-4483351 | Journal: *Plants* (MDPI)

This file contains the supporting material referenced in the revised manuscript: Table S1, the derivation audit trail for the zone-specific stress fractions of Table 3; Table S2, a diagnostic consistency check on those fractions; Text S1, the complete derivation of the photorespiration–stomatal coupling coefficient λ; and Figure S1, its sensitivity analysis. Reference numbers correspond to the reference list of the revised manuscript.

Table S1. Derivation audit trail for the zone-specific stress fractions

The protocol is described in Section 4.3 of the manuscript. Step 1 fixes the admissible range for each fraction from the process literature; Step 2 assigns a zone summer-stress index from elevation and precipitation; Step 3 adjusts for the contrasting drought strategies of the two species; Step 4 checks the result against dendroclimatic and process-model evidence. The columns below record, for each of the twelve zone × species units, the evidence invoked, what that evidence establishes, the transformation applied, and the fraction adopted in Table 3.

Step 1 — Process bounds applied to every zone. For the photorespiratory fraction: photorespiratory CO_2 release is roughly one quarter of net carboxylation in a well-watered C_3 leaf at 25 °C [9,11]; the theoretical efficiency loss approaches 48% at 30 °C at present-day CO_2 [11]; and the published range for forest canopies under combined heat and drought is 25–40% of GPP [10,11]. The admissible interval adopted is therefore $f_{PR} \in [0.10, 0.25]$. For the stomatal fraction, the interval adopted is $f_{ST} \in [0.07, 0.25]$, bounded below by zones in which summer water deficit is not growth-limiting and above by the assimilation suppression reported for Mediterranean pines during the July–August window [13–15].

Zone × species	Evidence	What the evidence establishes	Transformation applied	f_{PR}	f_{ST}
P. brutia · Mediterranean Coast	[15,16]	VPD exerts stronger control on maximum photosynthetic capacity than the land-surface moisture index; at the highest-VPD sites, assimilation is concentrated in spring, and the summer is effectively forgone [15].	Warmest and most illuminated zone in the estate; canopy remains lit and warm while assimilation is curtailed, so the photorespiratory fraction is set at the top of the Step-1 interval, and the stomatal fraction is high but not maximal.	0.25	0.20
P. brutia · Mediterranean Mid	[16,19]	The realised photosynthetic optimum lies below the biochemical optimum because closure pulls the effective T_{opt} downward [16]; productivity tracks site quality along the Taurus elevational gradient [19].	Mid-elevation buffering of afternoon temperature and VPD; both fractions reduced from the coastal values in proportion to the elevation band.	0.18	0.14
P. brutia · Aegean	[15]	Same VPD-dominated seasonal trajectory as the Mediterranean populations, at slightly lower summer temperature.	Interpolated between the Mediterranean Coast and Mediterranean Mid settings.	0.20	0.16
P. brutia · Marmara	[16]	Process-model sensitivity of GPP and NEP to temperature	Maritime, humid zone: summer thermal and evaporative stress is buffered	0.13	0.11

Zone × species	Evidence	What the evidence establishes	Transformation applied	f_{PR}	f_{ST}
		and water along gradients [16].	by proximity to the Sea of Marmara and by higher summer cloud cover, so both fractions are set well below the elevation-implied value.		
P. brutia · Interior Transitional	[18,19]	Drought sensitivity of radial growth is greatest at the dry, continental margins of the range [18,19].	Lowest precipitation of the *P. brutia* zones; stomatal fraction raised to equal the photorespiratory fraction.	0.20	0.20
P. brutia · Black Sea (isolated)	[16]	Process-model gradient behaviour [16].	Wettest zone in the estate and strongly maritime; both fractions set at the floor of the Step-1 intervals.	0.10	0.07
P. nigra · Central Anatolia	[18]	May–June precipitation is the principal limiting variable for radial growth in the eastern Mediterranean range of the species [18].	Driest zone in the estate; stomatal fraction set at the ceiling of the Step-1 interval, photorespiratory fraction moderate because elevation limits canopy temperature.	0.15	0.25
P. nigra · Mediterranean Mts. (Taurus)	[14]	Severe drought causes hydraulic failure and carbon starvation in declining stands, as evidenced by isotopic data showing suppressed conductance throughout the season [14].	Higher precipitation than the Anatolian interior; both fractions are intermediate.	0.16	0.14
P. nigra · Aegean Hinterland	[18]	Growth–climate sensitivity intermediate between the coastal and continental extremes [18].	Interpolated between the Taurus and Central Anatolia settings.	0.18	0.17
P. nigra · Marmara / NW Anatolia	[16]	Process-model gradient behaviour [16].	Maritime buffering for the *P. brutia* Marmara zone; both fractions set to the same values.	0.13	0.11
P. nigra · Eastern Anatolia	[14]	Drought-induced decline mechanisms as above [14], at the cold, high-elevation margin.	At high elevation, canopy temperature is lower, so the photorespiratory fraction is low; low precipitation keeps the stomatal fraction high.	0.12	0.22
P. nigra · Black Sea Transition	[16]	Process-model gradient behaviour [16].	Wet, maritime-influenced transition zone; both fractions at the floor of the Step-1 intervals.	0.10	0.07

Each fraction carries a ±25% plausibility interval, propagated through the Monte Carlo analysis of Section 5.5. The evidence column identifies the sources that constrain each setting; it does not imply that those sources report ecoregion-scale GPP-loss fractions directly, since none do.

Table S2. Diagnostic consistency check on the adopted fractions

Step 2 of the protocol maps a normalised summer-stress index onto the Step-1 intervals. To test how far the adopted fractions can be recovered from that mapping alone, a warmth index was computed as the elevation-band midpoint normalised across the twelve zones and inverted, and a dryness index as the precipitation midpoint normalised and inverted. The mechanical mapping is then $f_{PR} = 0.10 + 0.15 \times$ warmth and $f_{ST} = 0.07 + 0.18 \times$ dryness.

Zone × species	Elev. mid (m)	MAP mid (mm)	Warmth	Dryness	f_{PR} mapped	f_{PR} adopted	f_{ST} mapped	f_{ST} adopted	Deviation
P. brutia · Mediterranean Coast	250	900	1.00	0.29	0.25	0.25	0.12	0.20	f_{ST} raised
P. brutia · Mediterranean Mid	750	850	0.60	0.36	0.19	0.18	0.13	0.14	consistent
P. brutia · Aegean	450	700	0.84	0.57	0.23	0.20	0.17	0.16	consistent
P. brutia · Marmara	300	750	0.96	0.50	0.24	0.13	0.16	0.11	both lowered
P. brutia · Interior Transitional	850	450	0.52	0.93	0.18	0.20	0.24	0.20	consistent
P. brutia · Black Sea	250	1100	1.00	0.00	0.25	0.10	0.07	0.07	f_{PR} lowered
P. nigra · Central Anatolia	1300	400	0.16	1.00	0.12	0.15	0.25	0.25	consistent
P. nigra · Mediterranean Mts.	1400	700	0.08	0.57	0.11	0.16	0.17	0.14	consistent
P. nigra · Aegean Hinterland	1050	550	0.36	0.79	0.15	0.18	0.21	0.17	consistent
P. nigra · Marmara / NW	850	650	0.52	0.64	0.18	0.13	0.19	0.11	both lowered
P. nigra · Eastern Anatolia	1500	475	0.00	0.89	0.10	0.12	0.23	0.22	consistent
P. nigra · Black Sea Transition	1150	800	0.28	0.43	0.14	0.10	0.15	0.07	both lowered

Elevation and precipitation midpoints are taken from Tables 1 and 2 of the manuscript. Shaded rows are those in which the adopted fraction departs materially from the mechanical mapping. Across the twelve zones, the mapping recovers the stomatal fractions well (Pearson r = 0.71, mean absolute residual 0.035), but the photorespiratory fractions only weakly (r = 0.28, mean absolute residual 0.044).

The pattern in the residuals is systematic rather than random and interpretable. The four zones in which the adopted fractions fall well below the mechanical mapping — Marmara, Black Sea, Marmara / NW Anatolia and Black Sea Transition — are precisely the maritime and humid zones of the estate. In these zones, the elevation band is a poor proxy for canopy thermal load, because proximity to the sea, higher summer cloud cover and lower vapour-pressure deficit buffer the midday temperature maximum that drives the photorespiratory penalty. Elevation alone does not capture that buffering, and the mapping consequently overstates the photorespiratory fraction wherever a low-elevation zone is also maritime. The single zone in which the adopted stomatal fraction exceeds the mapping, the Mediterranean Coast, is the converse case: the dryness index is computed from annual precipitation, which is comparatively high there, whereas the limiting quantity is the depth of the July–August soil-moisture minimum rather than the annual total.

Text S1. Derivation of the photorespiration–stomatal coupling coefficient λ

S1.1 Statement of the problem. Equation (2) of the manuscript writes the effective photorespiratory fraction as $f_{PR,eff} = f_{PR}\ (1 + \lambda\ f_{ST})$, in which f_{PR} is the fraction of gross carboxylation lost to photorespiration under well-watered conditions and λ measures how far stomatal closure inflates that loss. The submitted version of the manuscript used the same term as $f_{PR}(1 + 0.5\ f_{ST}/0.20)$, which is algebraically identical to $\lambda = 0.5/0.20 = 2.5$, but which introduced a 20% stomatal loss as an unexplained reference state. The reformulation removes that reference state. This section derives λ from Rubisco kinetics and leaf energy balance rather than assuming it.

S1.2 Photorespiratory fraction in terms of chloroplastic CO{s}2{/s}. In the Farquhar–von Caemmerer–Berry formulation [27], the ratio of oxygenation to carboxylation is set by the CO_2 compensation point in the

absence of dark respiration, Γ*, relative to the chloroplastic CO_2 concentration, C_c. The photorespiratory CO_2 release per unit gross carboxylation is

$$f_{PR} = \Gamma^* / C_c \quad \text{(S1)}$$

and the net assimilation rate under Rubisco limitation is

$$A(C_c) = V_{cmax} (C_c - \Gamma_*) / (C_c + K_m), \qquad K_m = K_c(1 + O / K_o) \quad \text{(S2)}$$

Temperature responses for Γ*, K_c and K_o are taken from Bernacchi et al. [9], with 25 °C values $\Gamma_* = 42.75$ µmol mol⁻¹, $K_c = 404.9$ µmol mol⁻¹ and $K_o = 278.4$ mmol mol⁻¹, each scaled by an Arrhenius function with activation energies 37.83, 79.43 and 36.38 kJ mol⁻¹, respectively.

S1.3 Two amplification routes. Stomatal closure raises Γ_*/C_c by two multiplicative routes, and λ is the product of the two.

The **diffusive route** arises because assimilation under closure is diffusion-limited, so C_c must fall. Equation (S2) fixes how far it falls for a prescribed fractional reduction in assimilation: if closure reduces A by a fraction f_{ST}, then the stressed chloroplastic concentration C_c' solves $A(C_c') = (1 - f_{ST})\, A(C_c)$. The amplification is C_c / C_c'. This route is indifferent to whether the additional resistance is stomatal or mesophyll, which matters because mesophyll conductance also declines under water stress.

The **thermal route** arises because closure suppresses latent cooling. Needle temperature rises above air temperature, and Γ* rises with it under the temperature function of [9]. Writing the excess needle temperature as ΔT_{leaf}, the amplification is $\Gamma_*(T_{air} + \Delta T_{leaf}) / \Gamma_*(T_{air})$. We assume ΔT_{leaf} scales linearly with the degree of closure, calibrated as 4 °C at $f_{ST} = 0.20$.

Combining the two,

$$1 + \lambda f_{ST} = [C_c / C_c'] \times [\Gamma_*(T_{air} + \Delta T_{leaf}) / \Gamma_*(T_{air})] \quad \text{(S3)}$$

S1.4 Worked example. At $T_{air} = 28$ °C, $C_a = 400$ µmol mol⁻¹, $C_i/C_a = 0.65$, $g_m = 0.15$ mol m⁻² s⁻¹ bar⁻¹ and $A = 10$ µmol m⁻² s⁻¹, the unstressed chloroplastic concentration is $C_c = 260 - 67 = 193$ µmol mol⁻¹. A 20% reduction in assimilation requires $C_c' = 159$ µmol mol⁻¹, a diffusive amplification of 1.198. A 4 °C rise in needle temperature raises Γ* by a factor of 1.219. The product is 1.461, so from Equation (S3), $\lambda = (1.461 - 1) / 0.20 = 2.30$.

Evaluation condition	Diffusive	Thermal	Combined	λ
28 °C, $\Delta T_{leaf} = 4$ °C	1.198	1.219	1.461	2.30
32 °C, $\Delta T_{leaf} = 4$ °C	1.173	1.213	1.423	2.12
36 °C, $\Delta T_{leaf} = 4$ °C	1.149	1.207	1.387	1.94
28 °C, $\Delta T_{leaf} = 5$ °C	1.198	1.278	1.531	2.67
28 °C, $\Delta T_{leaf} = 3$ °C	1.198	1.161	1.391	1.95
Diffusive route only ($\Delta T_{leaf} = 0$)	1.15–1.20	1.000	1.15–1.20	0.75–0.99
Envelope, T 28–36 °C, ΔT_{leaf} 3–5 °C	—	—	—	1.45–2.85

Table S3. Derivation of λ at $f_{ST} = 0.20$ under the reference condition of Section S1.4. The diffusive route alone yields λ below unity; the thermal route roughly doubles it. The value adopted in the manuscript, $\lambda = 2.5$, lies inside the envelope and corresponds to a needle warming of approximately 4.5–5 °C at air temperatures of 28–32 °C.

S1.5 Choice of evaluation point. The coupling term is active only when $f_{ST} > 0$, that is, during the summer stress window in which stomatal closure and needle warming are both at their seasonal maximum. It is therefore evaluated under summer-window conditions rather than annual-mean conditions, which is why an air temperature of 28–32 °C and a needle warming of 4–5 °C are appropriate settings. Evaluating at annual-mean conditions would, by construction, understate the coupling.

S1.6 Consequence of the choice. The result is not sensitive to the value adopted. In the Monte Carlo analysis, λ is drawn from a triangular distribution over the derived envelope; its Spearman rank correlation with the

annual economic deficit is 0.09, ranking it seventh of the eight parameters tested. Setting λ = 0, which removes the coupling entirely, lowers the weighted NPP reduction from 37.9% to 31.6% and the forgone sequestration from 64.3 to 53.5 Mt CO_2 yr^{-1}. Adopting the envelope midpoint λ = 2.05 instead of 2.5 results in a 36.8% reduction, 62.3 Mt CO_2 yr^{-1}, and an annual deficit of US$4.20 billion on the gross basis. The coupling is therefore material to the estimate's magnitude, but no conclusion of the study depends on the precise value.

Figure S1. Sensitivity of the coupling coefficient λ

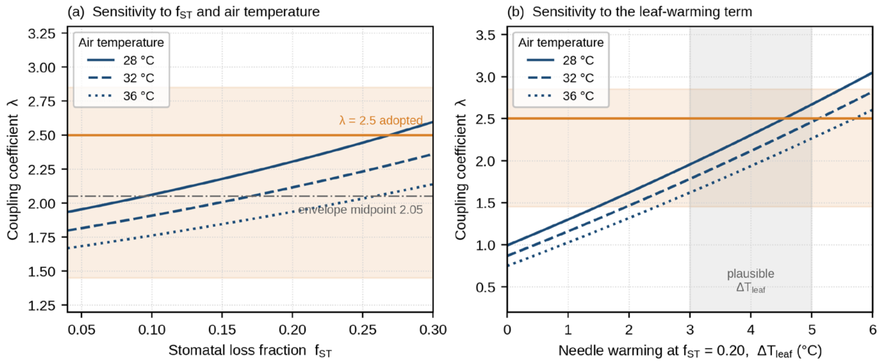

Figure S1. Sensitivity of the coupling coefficient λ derived in Text S1. (**a**) λ as a function of the stomatal loss fraction f_{ST} at three air temperatures, with the needle-warming term fixed at 4 °C when f_{ST} = 0.20 and scaling linearly. (**b**) λ as a function of the assumed needle warming ΔT_{leaf}, evaluated at f_{ST} = 0.20. The shaded horizontal band is the derived envelope λ = 1.45–2.85 used as the Monte Carlo range; the solid horizontal line is the value adopted in the manuscript; the vertical band in (**b**) marks the plausible range of needle warming for a closed conifer canopy under summer stress. λ falls as air temperature rises, because the Michaelis–Menten term K_m rises faster than Γ_* over this interval, so a given fractional reduction in assimilation requires a proportionally smaller drawdown of chloroplastic CO_2.

Reference numbers cited above correspond to the reference list of the revised manuscript. The model code and the scripts generating Tables S2 and S3 and Figure S1 are available from the corresponding author upon reasonable request.
